# The Molecular Pathogenesis and Clinical Implications of Hepatocellular Carcinoma

**DOI:** 10.4061/2011/818672

**Published:** 2011-12-04

**Authors:** Makoto Meguro, Toru Mizuguchi, Masaki Kawamoto, Koichi Hirata

**Affiliations:** Department of Surgical Oncology and Gastroenterological Surgery, Sapporo Medical University School of Medicine, S-1, W-16, Chuo-Ku, Sapporo, Hokkaido 060-8543, Japan

## Abstract

The prognosis of hepatocellular carcinoma (HCC) is affected by tumoral factors and liver functions; therefore it is often difficult to select the appropriate therapeutic methods for HCC. Recently, two global phase III trials showed that sorafenib, which is a tyrosine kinase inhibitor, improved the prognosis of patients with advanced HCC. As a new therapeutic strategy for HCC, sorafenib is expected to expand the indication for HCC in the future. However, it alone is insufficient for the molecular-targeted treatment of HCC because the signaling pathway exists not only in cancer cells but also in normal cells. Recently, cancer stem cells (CSCs) have attracted attention as a novel therapeutic target for HCC. There is now much evidence that stem cell properties such as self-renewal, unlimited proliferation, and differentiation are highly relevant to cancer recurrence and the drug resistance of HCC. In this review, we describe the molecular pathogenesis and the current state and future development of molecular- and CSC-therapeutic targeted agents for HCC, citing various reports.

## 1. Introduction

**Figure 1:**
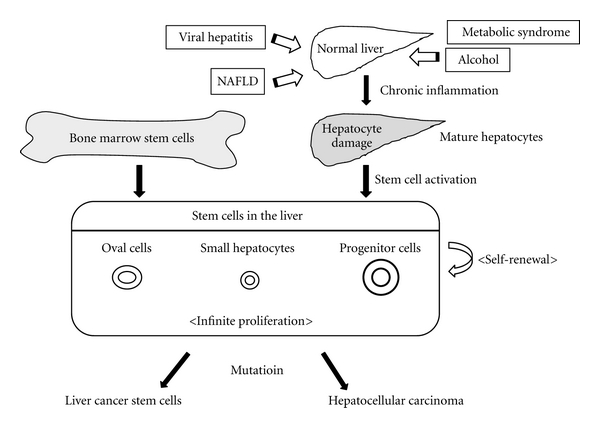
The etiology of HCC has been reported to be related to a variety of diseases such as viral hepatitis, alcoholic hepatitis, nonalcoholic fatty liver disease (NAFLD), and metabolic syndrome including diabetes mellitus. Regeneration of damaged hepatocytes reveals the activation of stem cells. The abilities of self renewal and infinite proliferation are closely related to the development of hepatocellular carcinoma (HCC). Stem cells in the liver are divided into several types, including oval cells, small hepatocytes, and progenitor cells. HCC cells and liver cancer stem cells could derive from mutation of these stem cells. The origin of the stem cells could be from either mature hepatocytes or bone marrow cells.

Hepatocellular carcinoma (HCC) is the sixth most common malignant disease worldwide and the third greatest cause of cancer-related death [1]. The etiology of HCC has been reported to be related to a variety of diseases such as viral hepatitis [2, 3], alcoholic hepatitis [4], nonalcoholic fatty liver disease (NAFLD) [5, 6], and metabolic syndrome including diabetes mellitus [7, 8]. The sequences from chronic hepatitis and liver cirrhosis cause de novo HCC [9] (Figure 1). HCC is considered to have increased invasiveness with malignant transformation and metastatic potential [10, 11]. Therefore, it is difficult to select the proper management of the disease. 

**Figure 2:**
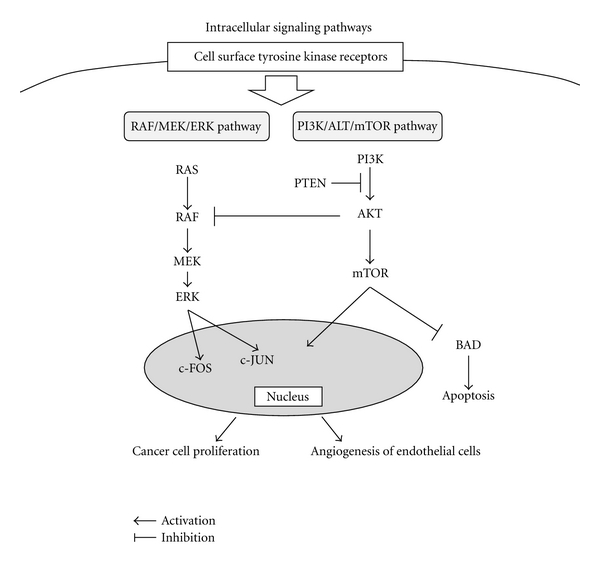
The RAF/MEK/ERK and the PI3K/AKT/mTOR signaling pathways are shown. Proangiogenic and proliferative growth factors activate the RAF/MEK/ERK pathway. The small GTPase RAS and the serine/threonine kinase RAF are the key molecular signal regulators. Intermediate signaling is regulated by MEK, which is responsible for phosphorylating and activating the final downstream signaling ERK molecules. ERK regulates cellular activity, indirect inducers of gene expression, and transcription factors in the AP-1 family such as c-JUN and c-FOS and cell cycle-related kinases. Binding of these growth factors to their receptors also activates PI3K, which subsequently produces the lipid second messenger, and in turn activates serine/threonine kinase AKT. Activated AKT also phosphorylates several cytoplasmic proteins, most notably mTOR. The activation of mTOR increases cellular proliferation, and inactivation of BAD decreases apoptosis and increases cell survival. This pathway is negatively regulated by the phosphatase and tensin homolog deleted on chromosome 10 (PTEN), which targets the lipid products of PI3K for dephosphorylation.

The clinical therapy for HCC includes various modalities such as liver resection [12], liver transplantation [13], transarterial chemoembolization (TACE) [14], percutaneous ethanol injection therapy (PEIT) [15], radiofrequency ablation (RFA) [16], and chemotherapy including molecular-targeted therapy [17]. However, the high recurrence rate is a major concern after any treatment [18]. The reason for the high recurrence rate of HCC could be proliferation of epithelial cells and increased arterial vascularity [19, 20]. Furthermore, HCC cells themselves express various growth factors such as vascular endothelial growth factor (VEGF) [19], platelet-derived growth factor (PDGF) [20], epidermal growth factor (EGF) [21], fibroblast growth factor (FGF) [22], and insulin-like growth factor (IGF) [23], which induce cell proliferation in an autocrine fashion [24]. The receptors of these growth factors activate intracellular signals such as the RAF/MEK/ERK pathway [25] and the PI3K/AKT/mTOR pathway [26], which induce proliferation of both cancer and endothelial cells (Figure 2). These growth factors, including their intracellular molecules, are considered to be a specific target for HCC treatment.

 In clinical trials, sorafenib, which is an inhibitor of the VEGF receptor (VEGFR) and PDGF receptor (PDGFR), has been proven to have a survival benefit for nonresectable HCC compared a placebo in the best supportive care (BSC) setting [27, 28]. Phase III trials are ongoing to determine the survival benefit in patients who receive surgery or ablation [29]. On the other hand, the survival benefit of sorafenib has been limited to a few of months and other pathways need to be blocked to achieve longer survival. Side effects of sorafinib therapy are obstacles to continuation of the therapy because normal cells also express VEGFR and PDGFR, and sorafenib severely damages both normal and cancer cells. The development of anticancer drugs must focus on a specific target that is restricted to the cancer cells.

Cancer stem cells have been shown to be a target for cancer-specific therapy recently. The abilities of self-renewal and infinite proliferation are closely related to the nature of HCC development [30, 31]. Stem cells in the liver are divided into several cell types, including oval cells [32], small hepatocytes [33], and progenitor cells [34] (Figure 1). Liver cancer stem cells and HCC cells could derive from mutation of these stem cells. The origin of the stem cells could be either from mature hepatocytes or from bone marrow cells [35] (Figure 1). Thus, specific stem cell-based therapy could be another strategy to overcome the high recurrence rate of HCC [36]. We describe the molecular pathogenesis, molecular therapy, and stem cell-targeted therapy for HCC treatment in this review.

## 2. Role of Growth Factors and Angiogenesis in HCC

A specific pathological feature of HCC is high vascularity of the tumor. It is necessary to increase vascularity for cancer cell proliferation. VEGF, PDGF, EGF, FGF, and IGF, growth factors that facilitate high vascularity and cancer cell proliferation, are expressed not only in cancer cells but also in other surrounding cells. The high expression of the growth factors is also associated with tumor invasion and portal thrombosis [19, 20, 22].

Among the growth factors, high expression of EGF is related to differentiation and invasion by the cancer cells [21]. On the other hand, PDGF is related to metastatic behavior of HCC cells [20]. The proliferation of endothelial cells is important for metastasis and invasion by cancer cells. Therefore, growth factors play an important role in proliferation of cancer cells not only in an autocrine fashion but also in a paracrine fashion through surrounding cells [24]. In addition, antivascular factors decrease in the serum and tissue of HCC patients [37]. These reports indicated that specific growth factors can be targets for HCC treatment.

Based on the high vascularity of HCC, endothelial cells could be a target for HCC treatment. This approach could be promising because endothelial cells have normal cell physiology with stable genetic regulation, which can be easily manipulated by molecular target therapy.

## 3. Role of RAF/MEK/ERK Signaling Pathway in Developing HCC

Tyrosine kinase type receptors, such as VEGFR, PDGFR, EGFR, FGFR, and IGFR, activate intracellular RAS in the RAF/MEK/ERK pathway [19–23]. Subsequently, AP-1 family members such as c-JUN and c-FOS activate expression of various genes that induce cell proliferation and vasculogenesis [38] (Figure 2). The activation of the RAF/MEK/ERK pathway is related to the disease progression of HCC [39] and HBV-related HCC development [40]. Furthermore, HCV core protein activates RAF and is considered to play a role in the development of HCC [41].

RAS and RAF play important roles in which intracellular signals activate expression of various genes [42] (Figure 2). RAS activates RAF, which induces activation of MEK [43]. MEK activates ERK and its phosphorylation [43]. ERK regulates more than one hundred intracellular substrates directly and gene expression indirectly as cell kinase to activate transcription factors and cell cycle regulators [44, 45]. Activation of ERK is closely related to cancer cell proliferation and, thus, inhibition of ERK could have an anticancer effect [46].

## 4. Role of PI3K/AKT/mTOR Signaling Pathway in Developing HCC

The phosphatidylinositol-3 kinase (PI3K) pathway plays an important role in the proliferation and survival of cancer cells in various solid tumors, including HCC [26] (Figure 2). PI3K activates AKT, which is a lipid second messenger [42]. Subsequently, AKT phosphorylates various intracellular proteins, including mTOR [42]. The activation of mTOR induces cell proliferation and inactivates BAD [47]. Inactivation of BAD is important for cancer cells to survive by regulating apoptosis [47]. Inactivation of AKT has been shown to improve the antitumor effect of sorafenib in an animal model and thus it could have potential use for HCC treatment [48]. The PI3K pathway is regulated by phosphatase and tensin homolog deleted on chromosome 10 (PTEN) negatively and the expression of PTEN is suppressed in half of HCC cells clinically [49] (Figure 2). In fact, PTEN expression is suppressed by HBx protein in HBV hepatitis patients [23], and downregulation of PTEN is associated with tumor grade progression, tumor stage, and poor overall prognosis [49].

## 5. Molecular-Targeting Clinical Therapies and Trials for HCC

Sorafenib is an inhibitor of RAF that is activated by VEGF and PDGF [50–52]. It has been tested in phase III clinical trials, such as the SHARP trial [27] and Asia-Pacific trial [28] (Table 1). It improves overall survival in patients with advanced HCC compared to patients administered a placebo in the BSC setting. Other tyrosine kinase inhibitors were also tested in clinical trials. Sunitinib is an inhibitor of VEGFR and PDGFR [53] (Table 1). The clinical phase II trial of sunitinib for HCC treatment showed severe grade 3 to 4 side effects [53]. Therefore, the comparative study between sorafenib and sunitinive has ceased in April 2010.

Brivanib, erotinib, and TSU-68, which are inhibitors of growth factor receptors, have been clinically tested for advanced HCC patients as well. The response rates to single doses of sorafenib [27, 28], sunitinib [53, 54], brivanib [55], erlotinib [56], and TSU-68 [57] were 2.3–3.3%, 2.7–2.9%, 5.0%, 9.0%, and 8.6% respectively (Table 1). Phase II clinical trials using bevacizumab [58], a VEGFR inhibitor, and cetuximab [59], an EGFR inhibitor, had 13% and 0% RRs, respectively (Table 1).

Although the clinical results of single doses of these molecular-targeted agents were not totally satisfactory, bevacizumab and TSU-68 achieved 2-3% complete responses [57, 58] (Table 1). In addition, a phase II clinical trial of combination therapy using erlotinib and bevacizumab had a 25% response rate [60] (Table 1). Therefore, combination of these agents with appropriate management of the side effects could improve survival of patients with advanced HCC in the future.

Ongoing clinical trials using molecular-targeted agents for HCC are shown in Table 2. The STORM trial is a phase III clinical trial using a single dose of sorafenib alone for adjuvant therapy after liver resection and ablation [29]. The SILIUS trial is a phase III clinical trial using combination therapy with transarterial chemoinfusion (TACI) and sorafenib for advanced HCC patients. The SPACE trial and TACTICS trial are a phase II clinical trial using TACE and sorafenib for advanced HCC patients. The BRISK-PS trial is designed for second therapy using brivanib for advanced HCC patients resistant to sorafenib. The BRISK-TA employs adjuvant therapy using brivanib after TACE, and the BRISK-FL trial is a comparative clinical trial using sorafenib alone and brivanib alone. These BRISK trials are a phase III clinical trial. The clinical results of these molecular-targeted therapies have not all been published yet and we will need to interpret the results carefully in the future.

## 6. Molecular Markers of Cancer Stem Cells in HCC

Molecular markers of cancer stem cells are shared with either normal stem cells or progenitor cells. CD133 [61], CD90 [62], CD44 [62], and EPCAM [63, 64] have been shown to be markers for cancer stem cells in HCC patients (Table 3). These biomarkers can be useful to estimate the prognosis of HCC and they could be useful for specific targeted therapy for cancer cells.

 CD133 has been shown to be related to prognosis and metastasis in HCC patients [65]. Tumor proliferation was suppressed by anti-CD133 antibodies in a mouse model [66]. NSC74859 is a specific inhibitor of signal transducer and activator of transcription 3 (STAT3) activation and it decreases CD133-positive cells with suppression of cancer development [67]. In addition, CD133 cells increase in PTEN deleted mice [68], which indicates that PTEN can play an important role to regulate CD133-positive cancer stem cells. These basic studies suggest that CD133 can be a molecular target for HCC treatment.

 The ATP-binding cassette (ABC) transporters are a family of membrane transporters such as MDR1 [69], ABCG2 [70], and ABCC2 [71]. ABC transporters protect cells from cytotoxic agents to reduce the drug sensitivity. A combination of chemotherapy and inhibitors of ABC transporters could decrease not only the number of HCC cells but also that of cancer stem cells [72] (Table 3).

 CD90 is expressed in oval cells and progenitor cells and its expression is related to tumor development [73]. CD44 is a receptor of hyaluronate expressed on the cell surface and is often coexpressed with CD90 [62, 74]. Anti-CD44 treatment induces apoptosis in CD90-positive cells and thus CD44 plays an important role in the survival of cancer cells [75, 76]. EPCAM is another cell marker for progenitor cells and the direct target of the Wnt/beta catenin pathway [63, 77]. The knockdown of the Wnt/beta catenin by small interfering RNA (siRNA) of Wnt/beta catenin decreases the number of EPCAM-positive cells with suppression of tumor development and induces apoptosis [78]. CD13-positive cells are also potential cancer stem cells [79] (Table 3). These cells can be found in the peripheral areas of HCC after TACE treatment, which is considered to be related to tumor recurrence [80]. Furthermore, inhibitors of CD13 such as 24F can suppress the invasion and angiogenesis of HCC [81].

## 7. Summary

The molecular pathogenesis of HCC is important to understand the mechanism of tumor development as well as the high-recurrence behavior of HCC. Furthermore, each step of the molecular signals could be a target to control tumor progression. Further clinical studies using single agents and combination therapies need to be conducted for HCC treatment. The clinical benefits of cell-targeted therapy for cancer stem cells are eagerly awaited.

## Figures and Tables

**Table 1:** Study results of molecular-targeted agents for hepatocellular carcinoma.

Agent	Target	Year	Phase	Patients	CR (%)	PR (%)	SD (%)	RR (%)	PFS (months)	TTP (months)	OS (months)	Authors	Reference number
Sorafenib SHARP study versus placebo	RAF, VEGFR, PDGFR	2008	III	299	0	2.3	71.0	2.3	4.1	5.5	10.7	Llovet et al.	[27]
				303	0	0.7	67.0	0.7	4.9	2.8	7.9		
Sorafenib Asia-Pacific study versus placebo		2009	III	150	0	3.3	54.0	3.3	3.5	2.8	6.5	Cheng et al.	[28]
				76	0	1.3	27.6	1.3	3.4	1.4	4.2		

Sunitinib	VEGFR, PDGFR	2009	II	37	0	2.7	35.1	2.7	3.7	5.3	8.0	Faivre et al.	[53]
		2009	II	34	0	2.9	50.0	2.9	3.9	4.1	9.8	Zhu et al.	[54]

Brivanib	VEGFR, FGFR	2009	II	55	NR	NR	NR	5.0	NR	2.8	10.0	Raoul et al.	[55]

Erlotinib	EGFR	2005	II	38	0	9.0	50.0	9.0	NR	3.2	13.0	Philip et al.	[56]
Erlotinib + bevacizumab		2009	II	40	0	25.0	38.0	25.0	9.0	NR	15.7	Thomas et al.	[60]

TSU-68	VEGFR, PDGFR, FGFR	2008	I/II	35	2.9	5.7	42.9	8.6	NR	NR	NR	Kanai et al.	[57]

Bevacizumab	VEGF	2009	II	46	2	11.0	NR	13.0	6.9	NR	12.4	Siegel et al.	[58]

Cetuximab	EGF	2007	II	30	0	0.0	NR	0	1.4	NR	9.6	Zhu et al.	[59]

CR: complete response; PR: partial response; SD: stable disease; RR: response rate; PFS: progression-free survival; TTP: time to progression; OS: overall survival; NR: not reported.

**Table 2:** Ongoing clinical trials using molecular-targeted agents for hepatocellular carcinoma.

Acronym	Phase	Active arm	Control arm	Design of the clinical trials
STORM	III	Sorafenib	Placebo	Adjuvant therapy after resection or ablation
SILIUS	III	Sorafenib + TACI	Sorafenib	Combination therapy with hepatic arterial infusion chemotherapy (TACI)
SPACE	II	Sorafenib + TACE	Placebo + TACE	Combination therapy with transarterial chemoembolization (TACE)
TACTICS	II	Sorafenib + TACE	TACE alone	Combination therapy with transarterial chemoembolization (TACE)
BRISK-PS	III	Brivanib	Placebo	Second-line therapy in sorafenib-resistant HCC
BRISK-TA	III	Brivanib + TACE	Placebo + TACE	Combination therapy with transarterial chemoembolization (TACE)
BRISK-FL	III	Brivanib	Sorafenib	First-line clinical trial for brivanib versus sorafenib

**Table 3:** Markers for cancer stem cell of HCC in recent reports.

Markers	References
CD133	[61]
CD90	[62]
CD44	[62]
EPCAM	[63, 64]
ABC transporters	[72]
CD13	[79]

## References

[1] Parkin DM, Bray F, Ferlay J, Pisani P (2005). Global cancer statistics, 2002. *Ca-A Cancer Journal for Clinicians*.

[2] Kanwal F, Hoang T, Kramer JR (2011). Increasing prevalence of HCC and cirrhosis in patients with chronic hepatitis C virus infection. *Gastroenterology*.

[3] Xu YF, Yi Y, Qiu SJ (2010). PEBP1 downregulation is associated to poor prognosis in HCC related to hepatitis B infection. *Journal of Hepatology*.

[4] Petta S, Craxì A (2010). Hepatocellular carcinoma and non-alcoholic fatty liver disease: from a clinical to a molecular association. *Current Pharmaceutical Design*.

[5] Page JM, Harrison SA (2009). NASH and HCC. *Clinics in Liver Disease*.

[6] Qian Y, Fan JG (2005). Obesity, fatty liver and liver cancer. *Hepatobiliary and Pancreatic Diseases International*.

[7] El-Serag HB, Tran T, Everhart JE (2004). Diabetes increases the risk of chronic liver disease and hepatocellular carcinoma. *Gastroenterology*.

[8] Amarapurkar DN, Patel ND, Kamani PM (2008). Impact of diabetes mellitus on outcome of HCC. *Annals of Hepatology*.

[9] Morita K, Taketomi A, Soejima Y (2009). De novo hepatocellular carcinoma in a liver graft with sustained hepatitis C virus clearance after living donor liver transplantation. *Liver Transplantation*.

[10] Koike Y, Nakagawa K, Shiratori Y (2003). Factors affecting the prognosis of patients with hepatocellular carcinoma invading the portal vein—a retrospective analysis using 952 consecutive HCC patients. *Hepato-Gastroenterology*.

[11] Hsu HC, Sheu JC, Lin YH (1985). Prognostic histologic features of resected small hepatocellular carcinoma (HCC) in Taiwan: a comparison with resected large HCC. *Cancer*.

[12] Eguchi S, Kanematsu T, Arii S (2008). Comparison of the outcomes between an anatomical subsegmentectomy and a non-anatomical minor hepatectomy for single hepatocellular carcinomas based on a Japanese nationwide survey. *Surgery*.

[13] Hwang S, Lee SG, Belghiti J (2009). Liver transplantation for HCC: its role—eastern and western perspectives. *Journal of Hepato-Biliary-Pancreatic Surgery*.

[14] Bojanović A, Bosnjaković P, Stojanović M (2009). Precision TACE in therapy of primary malignant tumors of liver/hepatocellular carcinoma (HCC). *Acta Chirurgica Lugoslavica*.

[15] Mueller PR (1995). Percutaneous ethanol injection therapy (PEIT) for hepatocellular carcinoma (HCC). *Japanese Journal of Cancer Research*.

[16] Witczak-Malinowska K, Zadrozny D, Studniarek M (2003). Preliminary assessment of utility of radiofrequency ablation technique in treatment of primary hepatocellular carcinoma (HCC) in patients with hepatic cirrhosis. *Medical Science Monitor*.

[17] Shen YC, Hsu C, Cheng AL (2010). Molecular targeted therapy for advanced hepatocellular carcinoma: current status and future perspectives. *Journal of Gastroenterology*.

[18] Chun JM, Kwon HJ, Sohn J (2011). Prognostic factors after early recurrence in patients who underwent curative resection for hepatocellular carcinoma. *Journal of Surgical Oncology*.

[19] Li Q, Xu B, Fu L, Hao XS (2006). Correlation of four vascular specific growth factors with carcinogenesis and portal vein tumor thrombus formation in human hepatocellular carcinoma. *Journal of Experimental and Clinical Cancer Research*.

[20] Stock P, Monga D, Tan X, Micsenyi A, Loizos N, Monga SPS (2007). Platelet-derived growth factor receptor-*α*: a novel therapeutic target in human hepatocellular cancer. *Molecular Cancer Therapeutics*.

[21] Schiffer E, Housset C, Cacheux W (2005). Gefitinib, an EGFR inhibitor, prevents hepatocellular carcinoma development in the rat liver with cirrhosis. *Hepatology*.

[22] El-Assal ON, Yamanoi A, Ono T, Kohno H, Nagasue N (2001). The clinicopathological significance of heparanase and basic fibroblast growth factor expressions in hepatocellular carcinoma. *Clinical Cancer Research*.

[23] Feitelson MA, Pan J, Lian Z (2004). Early molecular and genetic determinants of primary liver malignancy. *Surgical Clinics of North America*.

[24] Sǎftoiu A, Ciurea T, Baniţǎ M (2004). Immunohistochemical assessment of angiogenesis in primary hepatocellular carcinoma. *Romanian Journal of Gastroenterology*.

[25] Huynh H, Nguyen TT, Chow KH, Tan PH, Soo KC, Tran E (2003). Over-expression of the mitogen-activated protein kinase (MAPK) kinase (MEK)-MAPK in hepatocellular carcinoma: its role in tumor progression and apoptosis. *BMC Gastroenterology*.

[26] Chen YL, Law PY, Loh HH (2005). Inhibition of P13K/Akt signaling: an emerging paradigm for targeted cancer therapy. *Current Medicinal Chemistry—Anti-Cancer Agents*.

[27] Llovet JM, Ricci S, Mazzaferro V (2008). Sorafenib in advanced hepatocellular carcinoma. *The New England Journal of Medicine*.

[28] Cheng AL, Kang YK, Chen Z (2009). Efficacy and safety of sorafenib in patients in the Asia-Pacific region with advanced hepatocellular carcinoma: a phase III randomised, double-blind, placebo-controlled trial. *The Lancet Oncology*.

[29] Printz C (2009). Clinical trials of note. Sorafenib as adjuvant treatment in the prevention of disease recurrence in patients with hepatocellular carcinoma (HCC) (STORM). *Cancer*.

[30] Jordan CT, Guzman ML, Noble M (2006). Cancer stem cells. *The New England Journal of Medicine*.

[31] Chiba T, Zheng YW, Kita K (2007). Enhanced self-renewal capability in hepatic stem/progenitor cells drives cancer initiation. *Gastroenterology*.

[32] Petersen BE, Bowen WC, Patrene KD (1999). Bone marrow as a potential source of hepatic oval cells. *Science*.

[33] Best DH, Coleman WB (2010). Liver regeneration by small hepatocyte-like progenitor cells after necrotic injury by carbon tetrachloride in retrorsine-exposed rats. *Experimental and Molecular Pathology*.

[34] Cogliati B, Aloia TPA, Bosch RV, Alves VAF, Hernandez-Blazquez FJ, Dagli MLZ (2010). Identification of hepatic stem/progenitor cells in canine hepatocellular and cholangiocellular carcinoma. *Veterinary and Comparative Oncology*.

[35] Ma B, Ren J, Jiang HF, Jia J (2008). Antitumor activities against hepatocellular carcinoma induced by bone marrow mesenchymal stem cells pulsed with tumor-derived exosomes. *Beijing Da Xue Xue Bao*.

[36] Klonisch T, Wiechec E, Hombach-Klonisch S (2008). Cancer stem cell markers in common cancers—therapeutic implications. *Trends in Molecular Medicine*.

[37] Dhar DK, Ono T, Yamanoi A (2002). Serum endostatin predicts tumor vascularity in hepatocellular carcinoma. *Cancer*.

[38] Ozanne BW, Spence HJ, McGarry LC, Hennigan RF (2007). Transcription factors control invasion: AP-1 the first among equals. *Oncogene*.

[39] Chen L, Shi Y, Jiang C-Y, Wei L-X, Wang Y-L, Dai G-H (2011). Expression and prognostic role of pan-Ras, Raf-1, pMEK1 and pERK1/2 in patients with hepatocellular carcinoma. *European Journal of Surgical Oncology*.

[40] Chen J, Siddiqui A (2007). Hepatitis B virus X protein stimulates the mitochondrial translocation of Raf-1 via oxidative stress. *Journal of Virology*.

[41] Shimotohno K, Watashi K, Tsuchihara K, Fukuda K, Marusawa H, Hijikata M (2002). Hepatitis C virus and its roles in cell proliferation. *Journal of Gastroenterology*.

[42] Avila MA, Berasain C, Sangro B, Prieto J (2006). New therapies for hepatocellular carcinoma. *Oncogene*.

[43] Roberts LR, Gores GJ (2005). Hepatocellular carcinoma: molecular pathways and new therapeutic targets. *Seminars in Liver Disease*.

[44] Sananbenesi F, Fischer A, Schrick C, Spiess J, Radulovic J (2002). Phosphorylation of hippocampal Erk-1/2, Elk-1, and p90-Rsk-1 during contextual fear conditioning: interactions between Erk-1/2 and Elk-1. *Molecular and Cellular Neuroscience*.

[45] Halaschek-Wiener J, Wacheck V, Kloog Y, Jansen B (2004). Ras inhibition leads to transcriptional activation of p53 and down-regulation of Mdm2: two mechanisms that cooperatively increase p53 function in colon cancer cells. *Cellular Signalling*.

[46] Schmitz KJ, Wohlschlaeger J, Lang H (2008). Activation of the ERK and AKT signalling pathway predicts poor prognosis in hepatocellular carcinoma and ERK activation in cancer tissue is associated with hepatitis C virus infection. *Journal of Hepatology*.

[47] Roberts LR, Gores GJ (2006). Emerging drugs for hepatocellular carcinoma. *Expert Opinion on Emerging Drugs*.

[48] Chen K-F, Chen H-L, Tai W-T (2011). Activation of phosphatidylinositol 3-kinase/Akt signaling pathway mediates acquired resistance to sorafenib in hepatocellular carcinoma cells. *Journal of Pharmacology and Experimental Therapeutics*.

[49] Hu TH, Huang CC, Lin PR (2003). Expression and prognostic role of tumor suppressor gene PTEN/MMAC1/TEP1 in hepatocellular carcinoma. *Cancer*.

[50] Takezawa K, Okamoto I, Yonesaka K (2009). Sorafenib inhibits non-small cell lung cancer cell growth by targeting B-RAF in KRAS wild-type cells and C-RAF in KRAS mutant cells. *Cancer Research*.

[51] Zhu AX (2008). Development of sorafenib and other molecularly targeted agents in hepatocellular carcinoma. *Cancer*.

[52] Höpfner M, Schuppan D, Scherübl H (2008). Growth factor receptors and related signalling pathways as targets for novel treatment strategies of hepatocellular cancer. *World Journal of Gastroenterology*.

[53] Faivre S, Raymond E, Boucher E (2009). Safety and efficacy of sunitinib in patients with advanced hepatocellular carcinoma: an open-label, multicentre, phase II study. *The Lancet Oncology*.

[54] Zhu AX, Sahani DV, Duda DG (2009). Efficacy, safety, and potential biomarkers of sunitinib monotherapy in advanced hepatocellular carcinoma: a phase II study. *Journal of Clinical Oncology*.

[55] Raoul JL, Finn RS, Kang YK (2009). An open-label phase II study of first- and second-line treatment with brivanib in patients with hepatocellular carcinoma (HCC). *Journal of Clinical Oncology*.

[56] Philip PA, Mahoney MR, Allmer C (2005). Phase II study of Erlotinib (OSI-774) in patients with advanced hepatocellular cancer. *Journal of Clinical Oncology*.

[57] Kanai F, Yoshida H, Tateishi R (2011). A phase I/II trial of the oral antiangiogenic agent TSU-68 in patients with advanced hepatocellular carcinoma. *Cancer Chemotherapy and Pharmacology*.

[58] Siegel AB, Cohen EI, Ocean A (2008). Phase II trial evaluating the clinical and biologic effects of bevacizumab in unresectable hepatocellular carcinoma. *Journal of Clinical Oncology*.

[59] Zhu AX, Stuart K, Blaszkowsky LS (2007). Phase 2 study of cetuximab in patients with advanced hepatocellular carcinoma. *Cancer*.

[60] Thomas MB, Morris JS, Chadha R (2009). Phase II trial of the combination of bevacizumab and erlotinib in patients who have advanced hepatocellular carcinoma. *Journal of Clinical Oncology*.

[61] Ma S, Chan KW, Hu L (2007). Identification and characterization of tumorigenic liver cancer stem/progenitor cells. *Gastroenterology*.

[62] Yang ZF, Ngai P, Ho DW (2008). Identification of local and circulating cancer stem cells in human liver cancer. *Hepatology*.

[63] Yamashita T, Budhu A, Forgues M, Xin WW (2007). Activation of hepatic stem cell marker EpCAM by Wnt-*β*-catenin signaling in hepatocellular carcinoma. *Cancer Research*.

[64] Inagawa S, Itabashi M, Adachi S (2002). Expression and prognostic roles of *β*-catenin in hepatocellular carcinoma: correlation with tumor progression and postoperative survival. *Clinical Cancer Research*.

[65] Song W, Li H, Tao K (2008). Expression and clinical significance of the stem cell marker CD133 in hepatocellular carcinoma. *International Journal of Clinical Practice*.

[66] Smith LM, Nesterova A, Ryan MC (2008). CD133/prominin-1 is a potential therapeutic target for antibody-drug conjugates in hepatocellular and gastric cancers. *British Journal of Cancer*.

[67] Lin L, Amin R, Gallicano GI (2009). The STAT3 inhibitor NSC 74859 is effective in hepatocellular cancers with disrupted TGF-*β* signaling. *Oncogene*.

[68] Rountree CB, Ding W, He L, Stiles B (2009). Expansion of CD133-expressing liver cancer stem cells in liver-specific phosphatase and tensle homolog deleted on chromosome 10-deleted mice. *Stem Cells*.

[69] Wakamatsu T, Nakahashi Y, Hachimine D, Seki T, Okazaki K (2007). The combination of glycyrrhizin and lamivudine can reverse the cisplatin resistance in hepatocellular carcinoma cells through inhibition of multidrug resistance-associated proteins. *International Journal of Oncology*.

[70] Hu C, Li H, Li J (2008). Analysis of ABCG2 expression and side population identifies intrinsic drug efflux in the HCC cell line MHCC-97L and its modulation by Akt signaling. *Carcinogenesis*.

[71] Kitisin K, Ganesan N, Tang Y (2007). Disruption of transforming growth factor-*β* signaling through *β*-spectrin ELF leads to hepatocellular cancer through cyclin D1 activation. *Oncogene*.

[72] Dean M, Fojo T, Bates S (2005). Tumour stem cells and drug resistance. *Nature Reviews Cancer*.

[73] Yang ZF, Ho DW, Ng MN (2008). Significance of CD90^+^ cancer stem cells in human liver cancer. *Cancer Cell*.

[74] Herrera MB, Bruno S, Buttiglieri S (2006). Isolation and characterization of a stem cell population from adult human liver. *Stem Cells*.

[75] Endo K, Terada T (2000). Protein expression of CD44 (standard and variant isoforms) in hepatocellular carcinoma: relationships with tumor grade, clinicopathologic parameters, p53 expression, and patient survival. *Journal of Hepatology*.

[76] Yang GH, Fan J, Xu Y (2008). Osteopontin combined with CD44, a novel prognostic biomarker for patients with hepatocellular carcinoma undergoing curative resection. *Oncologist*.

[77] Yamashita T, Forgues M, Wang W (2008). EpCAM and *α*-fetoprotein expression defines novel prognostic subtypes of hepatocellular carcinoma. *Cancer Research*.

[78] Zeng G, Apte U, Cieply B, Singh S, Monga SPS (2007). siRNA-mediated *β*-catenin knockdown in human hepatoma cells results in decreased growth and survival. *Neoplasia*.

[79] Haraguchi N, Ishii H, Mimori K (2010). CD13 is a therapeutic target in human liver cancer stem cells. *Journal of Clinical Investigation*.

[80] El-Serag HB, Rudolph KL (2007). Hepatocellular carcinoma: epidemiology and molecular carcinogenesis. *Gastroenterology*.

[81] Inagaki Y, Tang W, Zhang L, Du G, Xu W, Kokudo N (2010). Novel aminopeptidase N (APN/CD13) inhibitor 24F can suppress invasion of hepatocellular carcinoma cells as well as angiogenesis. *Bioscience Trends*.

